# An Atypical Presentation of Viral Myocarditis Masquerading as Non-ST-Elevation Myocardial Infarction (NSTEMI)

**DOI:** 10.7759/cureus.63768

**Published:** 2024-07-03

**Authors:** Talal Alomar, Katerina Liong, Jad Alsheikh, Deepti Boddupalli

**Affiliations:** 1 Internal Medicine, Creighton University School of Medicine, Phoenix, USA

**Keywords:** viral pericarditis, non-st segment elevation myocardial infarction (nstemi), viral myositis, infectious myocarditis, coxsackie b virus

## Abstract

Coxsackie B virus is primarily associated with fever, pharyngitis, and gastrointestinal symptoms, while myocarditis is rarely reported. We present a rare case of a 47-year-old male with a history of hypertension and obesity, who developed Coxsackie B virus-induced myositis, myocarditis, and polyarthralgia. The patient presented with worsening back pain radiating to his chest, migratory arthralgia, exertional dyspnea, and bilateral shoulder pain with arm weakness. Initial investigations revealed elevated creatinine kinase (CK) levels and troponin I, alongside a high white blood cell (WBC) count and C-reactive protein (CRP) levels.

Given the patient's symptoms and uptrending troponin without EKG changes, there was a high concern for non-ST-elevation myocardial infarction (NSTEMI), leading to initial treatment with aspirin and IV heparin. However, further questioning revealed a recent sore throat and contact with an ill family member, prompting investigations for an infectious etiology. A viral panel confirmed Coxsackie B virus infection. The patient made a full recovery with supportive care.

This case highlights the importance of considering viral causes, particularly the Coxsackie B virus, in patients presenting with muscle pain, cardiac symptoms, and joint pain. Comprehensive viral testing is crucial for early identification and appropriate management to prevent long-term complications. Understanding the mechanisms of Coxsackie B virus infection is essential for developing effective treatment strategies addressing both the viral infection and the inflammatory response.

## Introduction

Myalgias can often have a broad differential and may include weakness, swelling, pain, and tenderness. Because of muscles’ relative resistance to infection, infectious myositis is relatively uncommon. Furthermore, the most common viral cause of myositis is influenza [1]. However, other viruses, such as Coxsackie B virus, can also lead to this condition.

Myocarditis, the inflammation of the heart muscle, can result from infections, autoimmune diseases, and exposure to certain toxins or drugs. This condition can disrupt the heart's conduction system and impair its pumping efficiency, leading to arrhythmias and, in severe cases, heart failure. Symptoms of myocarditis often include chest pain, fatigue, shortness of breath, and fluid retention [2].

Coxsackie B virus, a member of the *Enterovirus *genus, is known for causing a spectrum of illnesses ranging from mild febrile conditions to severe diseases such as myocarditis and pancreatitis. This virus is primarily transmitted via the fecal-oral route but can also spread through respiratory droplets. While many infections are asymptomatic or result in mild symptoms, certain strains of Coxsackie B virus can lead to severe complications, particularly in individuals with compromised immune systems or underlying health conditions [2,3]. In addition to myocarditis, the Coxsackie B virus can cause pleurodynia (Bornholm disease), which presents as severe chest and abdominal pain, and aseptic meningitis, an inflammation of the membranes surrounding the brain and spinal cord [2]. Although hand, foot, and mouth disease is more commonly associated with the Coxsackie A virus, it can also be caused by the Coxsackie B virus [2,4].

Polyarthralgia, or pain in multiple joints, is another potential manifestation of Coxsackie B virus infection. This condition can significantly impact a patient’s quality of life, causing discomfort and difficulty in performing daily activities [4]. The simultaneous occurrence of myositis, myocarditis, and polyarthralgia due to the Coxsackie B virus is particularly rare and poses a complex clinical challenge.

## Case presentation

A 47-year-old male patient with a history of hypertension and obesity presented to the Emergency Department (ED) for a three-day history of worsening back pain radiating to his chest, migratory arthralgia, exertional dyspnea, and bilateral shoulder pain with arm weakness.

On arrival, the patient was tachycardic with a low-grade fever of 37.8°C. An electrocardiogram (EKG) showed sinus tachycardia. Labs were notable for elevated troponin, WBC count, creatinine kinase (CK), and C-reactive protein (Table 1). A negative CT angiogram (CTA) ruled out aortic dissection and pulmonary embolism (PE). CT C-spine and MRI were also ordered in light of the upper extremity weakness with no significant findings. The echocardiogram showed an ejection fraction of 60-65% with no abnormalities.

**Table 1:** Abnormal lab values on admission

Lab Tests	Results	Reference Ranges
Troponin 1 (on admission)	128 ng/dL	0-35 ng/dL
WBC	14,300/µL	4.8-10.8x10^3^/µL
Creatine kinase	558 Units/L	30-200 Units/L
C-reactive protein	232.1 mg/L	0.3-1 mg/L

**Figure 1:**
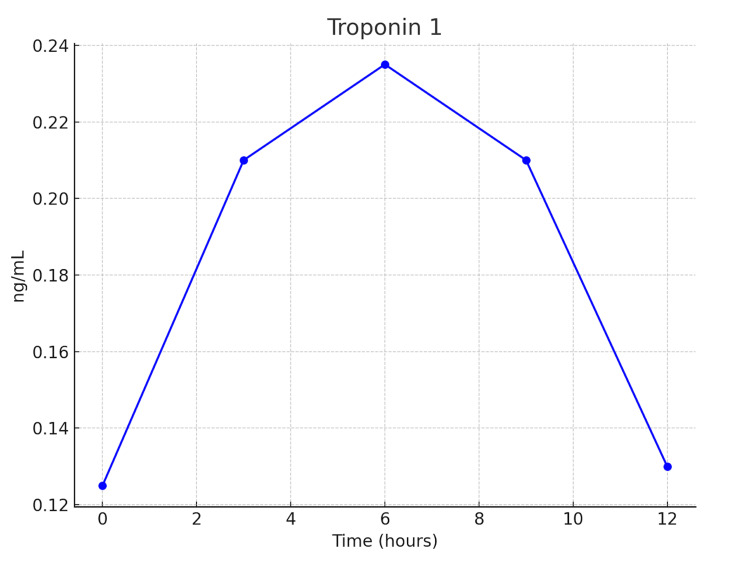
The graph of troponin levels during the first 14 hours of hospital stay.

Given the symptomatic findings as well as uptrending troponin without EKG changes, there was a high concern for non-ST-elevation myocardial infarction (NSTEMI). The patient was treated with aspirin and IV heparin. Troponin I began to downtrend after admission, and heparin was stopped (Figure 1). Cardiology indicated the minimal troponin elevation was likely not a primary ischemic event.

However, on further questioning, the patient noted a sore throat and chills and revealed that his wife had been sick a few weeks prior. Because of this, an infectious etiology was suspected, and blood cultures and respiratory viral panels were obtained. An extensive workup for autoimmune causes for his myositis was ordered. While these were pending, ampicillin/sulbactam and vancomycin were started.

Viral panel revealed Coxsackie B virus infection and was negative for autoimmune etiologies. Antibiotics were discontinued. The patient improved with two days of supportive treatment and was discharged in stable condition.

## Discussion

**Figure 2:**
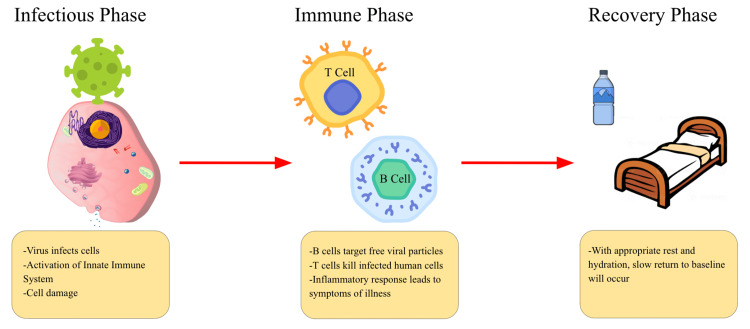
A diagram illustrating the phases of viral infection. Image Credits: Talal Alomar

Coxsackie B virus infections are typically associated with fever, pharyngitis, and gastrointestinal symptoms, but myocarditis occurs in only about 2% of cases [2,3]. The pathophysiology of Coxsackie B virus-induced myocarditis involves both direct viral invasion of myocardial cells and an immune-mediated response. The virus infects myocardial cells, leading to cell death and inflammation. The immune system may continue attacking myocardial cells even after the virus is cleared, exacerbating the damage [2,5]. This inflammation can disrupt the heart's conduction system, reduce its pumping efficiency, and lead to arrhythmias and heart failure (Figure 2).

Polyarthralgia due to Coxsackie B virus is rare, with only a few documented cases [4,6]. The virus can induce an inflammatory response in the joints, similar to other viral arthritis mechanisms, causing significant discomfort and difficulty in daily activities [4]. The simultaneous occurrence of myositis, myocarditis, and polyarthralgia in a single patient highlights the need for a thorough differential diagnosis when encountering such symptoms.

The rarity of this case lies in this simultaneous presentation, highlighting the diverse clinical spectrum of Coxsackie B virus infections. While myocarditis and myositis are serious conditions requiring prompt medical attention, the addition of polyarthralgia adds another layer of complexity to the patient’s clinical picture. This multifaceted presentation can pose diagnostic challenges, emphasizing the need for a thorough and systematic approach to patient evaluation.

Understanding the patterns of Coxsackie B virus infections and their associated complications is vital. While outbreaks of the Coxsackie B virus have been documented, the variability in clinical manifestations means that healthcare providers must maintain a high index of suspicion for this virus in patients presenting with atypical symptoms [3]. The interplay between environmental factors, host immunity, and viral virulence can influence the severity and range of symptoms, making each case unique.

In our patient, the presentation of myositis with elevated CK levels, myocarditis with elevated troponin levels, and polyarthralgia necessitated a broad differential diagnosis initially. The eventual identification of Coxsackie B virus as the causative agent underscores the importance of comprehensive viral testing in such cases. Early identification and appropriate antiviral and supportive treatments are crucial in managing the systemic effects of the virus and preventing long-term complications.

## Conclusions

In conclusion, this case highlights the importance of considering the Coxsackie B virus in the differential diagnosis of patients presenting with myositis, myocarditis, and polyarthralgia. It emphasizes the need for awareness of the diverse manifestations of this virus and the importance of a comprehensive and multidisciplinary approach to diagnosis and management. Given the potential severity of these conditions, timely and accurate diagnosis is essential for effective treatment and improved patient outcomes. The understanding of Coxsackie B virus’s mechanisms and its impact on various body systems continues to evolve, necessitating ongoing research and clinical vigilance.
